# Gastrointestinal complications are associated with a poor outcome in non-critically ill pneumonia patients

**DOI:** 10.1186/s12876-020-01537-z

**Published:** 2020-11-16

**Authors:** Chun-Ta Huang, Chun-Ming Hong, Yi-Ju Tsai, Wang-Huei Sheng, Chong-Jen Yu

**Affiliations:** 1 Department of Internal Medicine, National Taiwan University Hospital, No. 7, Chung-Shan South Road, Taipei, 100 Taiwan; 2 Graduate Institute of Clinical Medicine, National Taiwan University, Taipei, Taiwan; 3 Graduate Institute of Biomedical and Pharmaceutical Science, College of Medicine, Fu Jen Catholic University, New Taipei City, Taiwan

**Keywords:** Complication, Gastroenterology, Pneumonia, Prognosis, Readmission

## Abstract

**Background:**

Development of gastrointestinal (GI) complications is adversely associated with prognosis in the critically ill. However, little is known about their impact on the outcome of non-critically ill patients. In this study, we aimed to investigate the incidence of GI complications and their influence on prognosis of hospitalized pneumonia patients.

**Methods:**

Adult patients admitted with a diagnosis of pneumonia from 2012 to 2014 were included. Medical records were reviewed to obtain patients’ demographics, physical signs, comorbidities, laboratory results, clinical events, and the Confusion, Urea, Respiratory rate, Blood pressure and age ≥ 65 (CURB-65) score was calculated to assess the severity of pneumonia. GI complications, including bowel distension, diarrhea, GI bleeding and ileus, were evaluated during the first 3 days of hospitalization and their association with patient outcomes, such as hospital mortality and length of stay, was analyzed.

**Results:**

A total of 1001 patients were enrolled, with a mean age of 73.7 years and 598 (59%) male. Among them, 114 (11%) patients experienced at least one GI complication and diarrhea (5.2%) was the most common. The hospital mortality was 14% and was independently associated with an increase in the CURB-65 score (odds ratio [OR] 1.952 per point increase; 95% confidence interval [CI] 1.516–2.514), comorbid malignancy (OR 1.943; 95% CI 1.209–3.123), development of septic shock (OR 25.896; 95% CI 8.970–74.765), and the presence of any GI complication (OR 1.753; 95% CI 1.003–3.065).

**Conclusions:**

Compared to a critical care setting, GI complications are not commonly observed in a non-critical care setting; however, they still have a negative impact on prognosis of pneumonia patients, including higher mortality and prolonged length of hospital stay.

## Background

The gastrointestinal (GI) tract is the largest organ system of the human body and exerts a variety of physiologic functions during a normal state. Other than serving as a digestive conduit, the GI tract also plays an important role in immunomodulation, hormone control, fluid and electrolyte balance, and physical protection from ingested environmental threats [1–3]. During the period of critical illness, GI complications may occur as a result of diverse injurious mechanisms, such as hypoperfusion, ischemia–reperfusion injury and pro-inflammatory cytokine responses [1, 4, 5], and these complications are linked to increased mortality and morbidity among patients suffering from them [5, 6]. In this regard, occurrence of GI complications under a myriad of conditions may not be simply viewed as an innocent victim but can otherwise precipitate deleterious consequences.

Along this line, GI dysfunction has been proposed to be the motor of multiple organ dysfunction in critical illness although the pathophysiology involved (e.g., bacterial translocation, altered intestinal tight junctions, cytokine production and interaction with the gut microbiome) remains incompletely understood [3, 7–9]. However, despite of scientific interest in this issue, the clinical relevance of GI complications is still controversial with an unknown yet probably adverse impact on the outcome of patients. In addition, outside of intensive care settings, little if any is known about the effects of GI complications on clinical trajectories and prognosis of non-critically ill patients.

Therefore, in this study, we aimed to investigate the incidence of GI complications and their influence on patient outcomes under a non-critical care setting. Specifically, we chose to focus on the analysis of clinical information on patients with pneumonia because it is the most common cause of hospitalization and carries a significant risk of mortality around the globe. Moreover, by this way, we could include a more homogeneous patient population for comparisons of clinical presentation and disease severity between different groups of study subjects.

## Materials and methods

### Study settings and population

This retrospective observational study was conducted at a university-affiliated hospital in Taiwan. The protocol has been approved by the Research Ethics Committee of the National Taiwan University Hospital (201902005RIND) and written informed consent was waived because of the retrospective and non-interventional design of the study.

Adult patients ≥ 20 years of age admitted to the general medical wards between January 2012 and December 2014 were screened for eligibility in this study. Patients with an admission diagnosis of pneumonia were identified as the study subjects. The diagnosis of pneumonia was verified by a board-certified chest physician based on the overall medical records and radiologic findings [10]. For patients who were hospitalized twice or more during the study period, we included only the first hospitalization. Patients were excluded if they had an admission diagnosis other than pneumonia, were transferred from other facilities, or had missing data for calculating the severity score for pneumonia. In addition, patients with pre-existing GI disorders that may interfere the assessment of GI complications, such as inflammatory bowel disease, gastric/colorectal cancer, colostomy/ileostomy, and short bowel syndrome, were also excluded from the analysis.

### Data collection and definitions

Data retrieved on hospital admission included patient demographics, physical signs, comorbidities, laboratory testing results, and pertinent clinical events. Comorbidities of interest were chronic kidney disease, chronic obstructive pulmonary disease, coronary artery disease, diabetes mellitus, heart failure and malignancy [11, 12]. The Confusion, Urea, Respiratory rate, Blood pressure and age ≥ 65 (CURB-65) score was calculated to assess the severity of pneumonia [13]. GI complications were evaluated during the first 3 days of hospitalization and their definitions were described as follows: (a) bowel dilatation: radiologically confirmed bowel dilatation in any bowel segment; (b) diarrhea: loose or liquid stool three or more times per day; (c) GI bleeding: appearance of blood in vomited fluids, nasogastric aspirate or stool; (4) ileus: absence of stool for three or more days [2, 5]. Pertinent clinical events included development of septic shock and acute respiratory distress syndrome [14, 15], which were also assessed in the first 3 hospital days. In case that patients were rehospitalized after the index admission, the main reasons for rehospitalization were also obtained and categorized as infectious or non-infectious etiologies.

### Outcomes

Patients were followed up until death, 30 days after discharge or loss to follow-up, whichever came first. The primary outcome was the survival status at hospital discharge. Other outcomes of interest included length of stay during the hospitalization, and incidence of unscheduled readmission and time to readmission within 30 days after the index discharge among the hospital survivors.

### Statistical analysis

Continuous variables were presented as mean ± standard deviation or median (interquartile range) per data distribution, and were analyzed by the independent sample *t*-test or Mann–Whitney test, respectively. Categorical variables were reported as number (%) and were compared using the chi-square or Fisher's exact test, as appropriate. The logistic regression model was built to identify factors independently associated with hospital mortality in the multivariate analysis. Variables of laboratory testing were dichotomized by either the upper or lower limit of the reference range, as appropriate. All potentially confounding covariates were entered into the multivariate model without model selection. A two-tailed *P* value of < 0.05 indicated statistical significance. The statistical analyses were conducted by using the SPSS version 15.0 (SPSS Inc., Chicago, IL) software package.

## Results

**Fig. 1:**
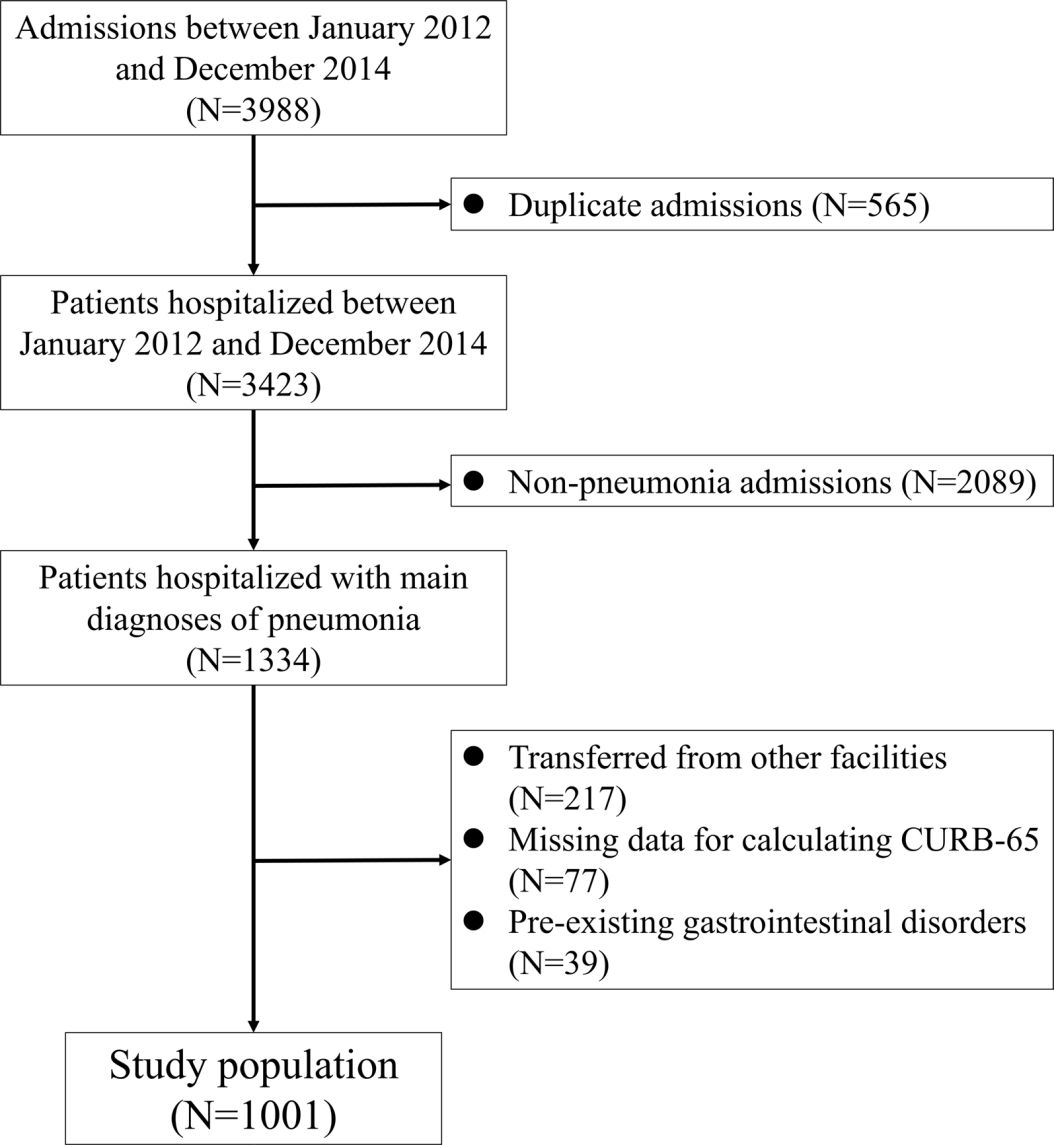
Study flow diagram. CURB-65, Confusion, Urea, Respiratory rate, Blood pressure and age ≥ 65

During the study period, a total of 1001 patients were included in this study (Fig. 1). The mean age of the study population was 73.7 ± 15.2 years and 598 (59%) of them were males (Table 1). On average, the CURB-65 score was 1.6 ± 1.0. The leading comorbidities were diabetes mellitus (28%), malignancy (19%) and chronic obstructive pulmonary disease (12%). Only few patients developed septic shock (2.3%) or acute respiratory distress syndrome (0.7%) in the first 3 days of hospitalization. The median length of hospital stay was 8 (5–12) days. The incidences of GI complications are presented in Table 2. Diarrhea (5.2%) was the most common complication and approximately 1 out of 9 patients (11%) experienced one or more GI complications.

**Table 1:** Baseline characteristics of the study population

Characteristic	Hospital survivors	Hospital non-survivors	*P* value
	N = 862	N = 139	
Age, years	73.2 ± 15.2	77.2 ± 14.7	0.003
Male sex	520 (60)	78 (56)	0.348
CURB-65 score	1.6 ± 1.0	2.1 ± 1.0	< 0.001
0	133 (15)	11 (7.9)	< 0.001
1	259 (30)	27 (19)	
2	322 (37)	51 (37)	
3	138 (16)	40 (29)	
4	10 (1.2)	10 (7.2)	
Comorbidity			
Diabetes mellitus	249 (29)	31 (22)	0.109
Malignancy	148 (17)	39 (28)	0.002
COPD	105 (12)	13 (9.4)	0.337
Coronary artery disease	99 (12)	16 (12)	0.993
Heart failure	84 (9.7)	15 (11)	0.701
Chronic kidney disease	56 (6.5)	10 (7.2)	0.758
Laboratory testing			
White blood cells, K/μL	11.0 ± 6.1	13.0 ± 8.2	0.006
Neutrophil-to-lymphocyte ratio	10.3 ± 10.6	15.6 ± 12.8	< 0.001
Albumin, g/dL	3.4 ± 0.3	3.2 ± 0.5	< 0.001
C-reactive protein, mg/dL	7.4 ± 4.8	8.6 ± 5.2	0.017
Events in the first 3 hospital days			
Septic shock	6 (0.7)	17 (12)	< 0.001
ARDS	6 (0.7)	1 (0.7)	0.999
Length of stay, days	8 (6–12)	8 (3–14)	0.025

ARDS, acute respiratory distress syndrome; COPD, chronic obstructive pulmonary disease; CURB, Confusion, Urea, Respiratory rate, Blood pressure

**Table 2:** Incidence of gastrointestinal (GI) complications within 3 days of hospital admission between survivors and non-survivors

Characteristic	All	Survivors	Non-survivors	*P* value
	N = 1001	N = 862	N = 139	
Diarrhea	52 (5.2)	42 (4.9)	10 (7.2)	0.252
Bowel dilatation	34 (3.4)	26 (3.0)	8 (5.8)	0.124
Ileus	25 (2.5)	19 (2.2)	6 (4.3)	0.143
GI bleeding	14 (1.4)	10 (1.2)	4 (2.9)	0.117
Any GI complication	114 (11)	90 (10)	24 (17)	0.019

There were 862 (86%) survivors and 139 (14%) non-survivors on hospital discharge (Table 1). The non-survivors were older (77.2 vs. 73.2 years; *P* = 0.003) and had a higher CURB-65 score (2.1 vs. 1.6; *P* < 0.001) than the survivors. Also, non-survivors were more likely to have comorbid malignancy (28% vs. 17%; *P* = 0.002) and experience septic shock (12% vs. 0.7%; *P* < 0.001) compared to survivors. Regarding laboratory testing, a higher white blood cell count (13.0 vs. 11.0 K/μL; *P* = 0.006), neutrophil-to-lymphocyte ratio (15.6 vs. 10.3; *P* < 0.001), and C-reactive protein value (8.6 vs. 7.4 mg/dL; *P* = 0.017) were observed in non-survivors than survivors. On the contrary, non-survivors had lower serum albumin levels than survivors (3.2 vs. 3.4 g/dL; *P* < 0.001). The length of hospital stay was longer in non-survivors compared to survivors (8 vs. 8 days in medians; *P* = 0.025). Regarding GI complications, no statistically significant differences were found in any single complication between the survivors and non-survivors (Table 2). However, non-survivors more often developed any of the GI complications compared to survivors (17% vs. 10%; *P* = 0.019).

Table 3 shows the logistic regression model incorporating patient demographics, comorbidities, laboratory results, clinical events, CURB-65 scores and the presence of GI complications for odds ratios (ORs) of hospital mortality in the pneumonia patients. Independent risk factors of mortality included an increase in CURB-65 scores (OR 1.952 per point increase; 95% confidence interval [CI] 1.516–2.514), comorbid malignancy (OR 1.943; 95% CI 1.209–3.123), development of septic shock (OR 25.896; 95% CI 8.970–74.765), and the presence of any GI complication (OR 1.753; 95% CI 1.003–3.065).

**Table 3:** Multivariate logistic regression analysis on hospital mortality

Parameter	Odds ratio	95% CI	*P* value
Age, ≥ 65 years	2.189	1.169–4.097	0.014
Male sex	0.677	0.451–1.017	0.060
CURB-65, per point increase	1.952	1.516–2.514	< 0.001
Comorbidity			
Diabetes mellitus	0.699	0.441–1.111	0.130
Malignancy	1.943	1.209–3.123	0.006
Heart failure	0.869	0.441–1.714	0.686
Coronary artery disease	1.028	0545–1.939	0.933
Chronic kidney disease	0.751	0.336–1.679	0.486
COPD	0.763	0.384–1.513	0.438
Laboratory testing			
White blood cells, > 9 K/μL	1.760	1.148–2.697	0.010
Neutrophil-to-lymphocyte ratio, > 3	2.445	1.016–5.883	0.046
Albumin, < 3.5 g/dL	2.421	1.554–3.771	< 0.001
C-reactive protein, ≥ 0.8 mg/dL	1.692	0.487–5.873	0.407
Events in the first 3 hospital days			
Septic shock	25.896	8.970–74.765	< 0.001
ARDS	0.760	0.083–6.954	0.808
Any GI complication	1.753	1.003–3.065	0.048

ARDS, acute respiratory distress syndrome; CI, confidence interval; COPD, chronic obstructive pulmonary disease; CURB, Confusion, Urea, Respiratory rate, Blood pressure; GI, gastrointestinal

In addition to an association with increased hospital mortality, the presence of GI complications in patients with pneumonia was also associated with a longer hospital stay (Table 4). Moreover, among the pneumonia patients surviving to hospital discharge, those with any of the GI complications during the first 3 days of hospitalization seemed more likely to be readmitted within 30 days of discharge than those without, and the majority (77%) of all these readmissions were ascribed to infectious diseases.

**Table 4:** Outcomes with regards to the presence of gastrointestinal (GI) complications

Characteristic	Without GI complications	With GI complications	*P* value
During hospitalization			
Number of patients	N = 887	N = 114	
Length of stay, days	8 (5–12)	10 (6–14)	0.038
Hospital mortality	115 (13)	24 (21)	0.019
Post-discharge^a^			
Number of patients	N = 666	N = 80	
Readmission	96 (14)	18 (23)	0.058
Infectious	76 (11)	12 (15)	
Non-infectious	20 (3.0)	6 (7.5)	
Time to readmission, days	14.3 ± 7.9	13.0 ± 8.5	0.537

^a^Follow-up status was available in 746 patients

## Discussion

The present study shows that, in a non-critical care setting, 1 out of 9 pneumonia patients would experience GI complications during the first 3 days of admission and these complications were associated with a higher odds of hospital mortality and a longer length of hospital stay. In addition, among the hospital survivors, those with GI complications were more likely to be rehospitalized within 30 days of the index discharge. Taken together, the findings indicate a deleterious role of GI complications in patients hospitalized for pneumonia and suggest that the importance of the GI tract in the pathophysiology of non-GI diseases should not be overlooked.

Several observational studies on critically ill patients consistently show an adverse impact of GI complications on the risk of mortality although the definitions of complications and their observation windows significantly differ between studies [5, 16–19]. Reintam-Blaser et al. reported that GI failure, defined as the presence of food intolerance, GI bleeding or ileus, was related to significantly higher mortality as well as prolonged lengths of intensive care unit (ICU) stay and mechanical ventilation [17]. The same study group later proposed a GI failure score, combining food intolerance and intra-abdominal hypertension into a 5-grade scoring system, and identified the GI failure score in the first 3 ICU days as an independent risk factor for ICU mortality [18]. They also demonstrated that during the first week of ICU admission, certain specific GI complications, including absent bowel sounds, GI bleeding and bowel distension, and the total number of GI complications were associated with 28-day mortality [5]. To the best of our knowledge, the current study is the first one to evaluate the prognostic role of GI complications in a less severe patient population. In line with the findings from ICU studies, development of GI complications in hospitalized pneumonia patients was associated with poor clinical outcomes, including hospital mortality and length of stay. In summary, our findings emphasize the importance of GI complications and extend their role from the critical to non-critical care settings.

Compared to ICU studies [5, 20, 21], the incidence of global GI complications in our study was quite low (59–80% vs. 11%). This may simply reflect the association between the severity of acute illness and risks of GI complications, as shown in two prospective observational studies [20, 22]. Zhang et al. showed a strong positive correlation between acute GI injury grading system and the acute physiology and chronic health evaluation (APACHE) II score [22]. Reintam-Blaser et al. found that the sequential organ failure assessment (SOFA) score on ICU admission was predictive of the development of GI symptoms [20]. In terms of individual GI complications, diarrhea, ileus, bowel distension and GI bleeding are commonly described in the literature [2], with a wide range of reported incidences (e.g., from 2 to 95% for diarrhea in the critically ill) [23]. Some of those complications, such as GI bleeding and bowel distension, have been reportedly associated with ICU or 28-day mortality [5, 24]. However, none of the GI complications alone was found to be predictive of hospital mortality in the current study, although the incidence of each complication was unanimously higher in non-survivors than in survivors (Table 2). Our study, probably limited by the study design and settings, may be underpowered to detect such a small, if any, effect size.

A major obstacle to studying GI complications is that the definitions are often varied, vague and elusive. The terminology can also be confusing. Terms, such as acute GI injury, food intolerance and GI failure, have been used interchangeably or to indicate different GI conditions across the literature [25]. Moreover, lack of objective measures of GI functions and validated biomarkers add to the complexity in this area [26]. In this regard, the Working Group on Abdominal Problems of the European Society of Intensive Care Medicine presented a consensus statement on the definitions and grading system of GI dysfunction for ICU patients [2]. Other investigators have provided evidence to support the prognostic significance of the acute GI injury grading system in the critically ill patients [19, 22]. In our study, however, this grading system was not utilized since high-grade GI injury was expected to occur sparsely among pneumonia patients admitted to general wards. Instead, simple, easily recognizable GI signs and symptoms were used to delineate the clinical profile of GI complications in our study population. As the first study in this field, our results should encourage researches to define explicit characteristics of GI conditions in non-critically ill patients.

An interesting finding in this study is that pneumonia patients with GI complications were readmitted within 30 days of discharge more often than those without, although the difference did not reach statistical significance. Great efforts have been put on identification of risk factors for readmission following hospitalization for pneumonia. Comorbidities, socioeconomic status, and so on play a pivotal role in this regard [27, 28]; however, to our knowledge, none of the studies identified the association between GI dysfunction during the index hospitalization and risks of readmission. Our finding may not be that unexpected but is pathophysiologically plausible. Gut origin of sepsis hypothesis proposes that translocation of potentially harmful bacteria across the intestinal barrier causes sepsis at distant sites [29]. Thus, GI dysfunction may render the host susceptible to infections and consequently result in rehospitalization. The observation that the majority of readmissions in this study could be ascribed to infectious diseases may partly support this hypothesis.

A number of limitations pertaining to this study should be considered. First, the incidence of GI complications can be underestimated since clinically insignificant events may not be reported by the patients or well documented in the medical records. However, important and influential GI conditions are unlikely to be missed, and our results are practically informative to clinicians in daily practice. Second, that only pneumonia patients were enrolled for analysis may limit the generalizability of the study findings to other non-critically ill populations. Nonetheless, inclusion of this non-GI disease alone is also the strength of our study because we were able to clearly delineate and analyze the effects of GI complications on patient outcomes. Undoubtedly, more studies are encouraged to validate our findings in other disease contexts. Third, the impact of pre-existing GI disorders on GI complications and the interactions between them remain to be elucidated. In our study, patients with pre-morbid GI diseases were excluded from analysis in order to distinctly define the development of GI complications. This is the advantage and also the disadvantage of our study design, and prospective, well-defined studies (e.g., meaningful changes in GI signs and symptoms) are required to solve these issues.

## Conclusions

GI complications are not that commonly observed outside of ICU settings; however, they still exert a negative impact on prognosis of pneumonia patients, including higher mortality and prolonged length of hospital stay. In addition, development of those complications in patients with pneumonia during the index hospitalization seems to be associated with the post-discharge outcome, namely, 30-day rehospitalization. Therefore, the findings altogether illustrate the important prognostic role of GI complications in patients with non-GI diseases under a non-critical care setting.

## Data Availability

The datasets used and/or analyzed during the current study are available from the corresponding author on reasonable request.

## Abbreviations

APACHE: Acute physiology and chronic health evaluation
CI: Confidence interval
CURB-65: Confusion, Urea, Respiratory rate, Blood pressure and age ≥ 65
GI: Gastrointestinal
ICU: Intensive care unit
OR: Odds ratio
SOFA: Sequential organ failure assessment
